# Medical News

**Published:** 1854-03

**Authors:** 


### THE MEDICAL EXAMINER.

PHILADELPHIA, MARCH, 1854.

### MEDICAL NEWS.

College of Physicians of Philadelphia.—At a meeting of the College, held February 1st, 1854, the following delegates were elected to the American Medical Association : Drs. F. G. Smith, J. D. Griscom, C. D. Meigs, John Neill, James M. Greene, S. L. Hollingsworth, J. B. Biddle, Lewis Rodman, G. B. Wood, Samuel Lewis, Robt. Bridges, Isaac Hays.

Philadelphia County Medical Society.—At a meeting of this Society, held January 18th, 1854, the following delegates to the American Medical Association, and to the State Medical Society were elected, viz.:

*To the American Medical Association.*—Drs. William Ashmead, Thomas F. Betton, Charles H. Bibighaus, Benjamin H. Coates, Levi Curtis, G. Emerson, Edward Hallowell, Edward Hartshorne, S. C. Huston, Nathan L. Hatfield, Alexander C. Hart, Wilson Jewell, Alfred L. Kennedy, William H. Klapp, John F. Lamb, Renè La Roche, Samuel Lewis, J. H. B. McLellan, W. S. W. Ruschenberger, Isaac Remington, R. H. Townsend, J. S. Zorns.

*To the State Medical Society* :—Drs. William Ashmead, T. E. Beesley, Thomas F. Betton, Charles H. Bibighaus, Benjamin H. Coates, D. Francis Condie, Isaac Comly, G. Emerson, James V. Emlen, George Fox, David Gilbert, Edward Hallowell, Isaac Hays, Henry Hartshorne, Nathan L. Hatfield, A. Helffenstein, Jacob Huckel, John H. Ingham, Samuel Jackson, Prof. Samuel Jackson, Drs. Benjamin S. Janney, Wilson Jewell, Alfred L. Kennedy, Joseph Klapp, William H. Klapp, S. Littell, Samuel Lewis, E. F. Leake, J. H. B. McLellan, George W. Norris, William B. Page, John M. Pugh, Isaac Remington, Lewis Rodman, W. S. W. Ruschenberger, Moses B. Smith, Robert P. Thomas, John F. Trenchard, Francis West, Caspar Wister, George B. Wood, Joshua H. Worthington, Thomas H. Yardley, Jacob S. Zorns.

Published by order, Robert P. Thomas.
Assistant Secretary.

The New Orleans Med. and Surg. Journal reports the following death from chloroform: A young female, whose great toe was about to be amputated by one of the visiting surgeons of the Charity Hospital, suddenly expired while under the influence of chloroform. It was some time before she could be brought fully under the effects of the anæsthetic; she finally, however, became completely insensible, and before the operation was concluded, she sank and rapidly expired in spite of the most strenuous and judicious efforts of several medical men present. The usual precautions were used in its administration, and no censure can justly be attached to the surgeon or his assistants for the untimely result of the case. A post-mortem was made by the Professor of Physiology in the University of Louisiana, and all the organs were found to be perfectly healthy.

---

Only one case of cholera was reported in London, during the week ending January 21st.

---

The Stethoscope, formerly edited by P. C. Gooch, M. D., has been purchased by the State Medical Society of Virginia. Its present editors are Drs. T. P. Atkinson, R. W. Haxall, J. Bolton, R. A. Lewis, A. T. B. Merritt and J. G. Cabell.

---

Dr. Joseph Parrish, the able editor of the New Jersey Medical Reporter for the last six years, has vacated his post in favor of S. W. Butler, M. D. Dr. P. will hereafter reside in Philadelphia. The Bibliographical department of the Reporter will be under the supervision of H. S. Patterson, M. D., of Philadelphia.

---

The catalogue of the Jefferson College gives 627 as the number of its students.

---

Obituary.—Dr. Lewis W. Chamberlayne, Professor of Materia Medica and Therapeutics, died in Richmond, Va., on the 28th of Jan., 1854.

---

GENERAL ORDER.

*Navy Department, February* 1, 1854.

1. Hereafter a Board of Surgeons in the Navy, shall assemble annually at such place as may be designated by the Department, about the close of the lecture seasons of the Colleges, for the examination and selection of candidates for admission into the Medical Corps of the Navy, and the examination of Assistant Surgeons who may be candidates for promotion.

2. The Board will select from the qualified candidates for admission, such a number of the best as may be necessary to meet the demands of the service for the following year.

3. As vacancies occur in the Medical Corps of the Navy, appointments will be made from the qualified candidates in the order of succession in which they may be named by the Board; but no appointment will be given to any such candidate who is over twenty-five years of age.

4. No qualified candidates will be held over for appointment after one year, but all such must be re-examined and take position in the class in which they are last examined.

5. Every candidate for admission will be examined, strictly and carefully, as to his physical capacity for the service, and the Board will make a separate report in each case, which will be forwarded *direct* to the Department, to be placed on file with the testimonials of the candidate.—This examination will precede that as to professional qualifications, and no candidate who is not physically qualified will be examined professionally.

6. In order that the relative position of Assistant Surgeons of the same date, who shall be examined for promotion at different times, may be more readily determined, a majority of the members of the Board will be selected, if practicable, from those who served on the next preceding Board.

7. Assistant Surgeons, who are candidates for promotion, shall present to the Board testimonials of correct deportment and habits of industry from the Surgeons with whom they have been associated on duty; also, a Journal of Practice, or Case Book, in their own hand-writing.—They are expected to be familiar with all the details of duty specified in the "Instructions for the government of Medical Officers."

J. C. Dobbin, Secretary of the Navy.

---

A Board of Naval Surgeons will assemble at the Naval Asylum, Philadelphia, on Monday, March 6th, 1854, for the examination of candidates for promotion, as well as for admission into the Medical staff of the Navy.

The Board will consist of Surgeon Thomas Dillard, President; James M. Greene, W. S. W. Ruschenberger, Members; and Passed Assistant Surgeon, A. A. Henderson, Recorder.

